# Bacterial otitis media in sub-Saharan Africa: a systematic review and meta-analysis

**DOI:** 10.1186/s12879-020-4950-y

**Published:** 2020-03-17

**Authors:** Tewodros Tesfa, Habtamu Mitiku, Mekonnen Sisay, Fitsum Weldegebreal, Zerihun Ataro, Birhanu Motbaynor, Dadi Marami, Zelalem Teklemariam

**Affiliations:** 1 Department of Medical Laboratory Sciences, College of Health and Medical Sciences, Haramaya University, P.O.Box, 235, Harar, Ethiopia; 2 Department of Pharmacology and Toxicology, School of Pharmacy, College of Health and Medical Sciences, Haramaya University, P.O.Box, 235, Harar, Ethiopia; 3 Department of Pharmaceutical Analysis, School of Pharmacy, College of Health and Medical Sciences, Haramaya University, P.O. Box, 235, Harar, Ethiopia

**Keywords:** Otitis media, Bacterial isolates, Antimicrobial resistance, Sub-Saharan Africa, Systematic review, Meta-analysis

## Abstract

**Background:**

Otitis media is inflammation of the middle ear, comprising a spectrum of diseases. It is the commonest episode of infection in children, which often occurs after an acute upper respiratory tract infection. Otitis media is ranked as the second most important cause of hearing loss and the fifth global burden of disease with a higher incidence in developing worlds like Sub-Saharan Africa and South Asia. Therefore, this systematic review is aimed to quantitatively estimate the current status of bacterial otitis media, bacterial etiology and their susceptibility profile in sub-Saharan Africa.

**Methods:**

A literature search was conducted from major databases and indexing services including EMBASE (Ovid interface), PubMed/MEDLINE, Google Scholar, ScienceDirect, Cochrane Library, WHO African Index-Medicus and others. All studies (published and unpublished) addressing the prevalence of otitis media and clinical isolates conducted in sub-Saharan Africa were included. Format prepared in Microsoft Excel was used to extract the data and data was exported to Stata version 15 software for the analyses. Der-Simonian-Laird random-effects model at a 95% confidence level was used for pooled estimation of outcomes. The degree of heterogeneity was presented with I^2^ statistics. Publication bias was presented with funnel plots of standard error supplemented by Begg’s and Egger’s tests. The study protocol is registered on PROSPERO with reference number ID: CRD42018102485 and the published methodology is available from http://www.crd.york.ac.uk/*CRD42018102485**.*

**Results:**

A total of 33 studies with 6034 patients were included in this study. All studies have collected ear swab/discharge samples for bacterial isolation. The pooled isolation rate of bacterial agents from the CSOM subgroup was 98%, patients with otitis media subgroup 87% and pediatric otitis media 86%. A univariate meta-regression analysis indicated the type of otitis media was a possible source of heterogeneity (*p*-value = 0.001). The commonest isolates were *P. aeruginosa* (23–25%), *S. aureus* (18–27%), *Proteus* species (11–19%) and *Klebsiella* species. High level of resistance was observed against Ampicillin, Amoxicillin-clavulanate, Cotrimoxazole, Amoxicillin, and Cefuroxime*.*

**Conclusion:**

The analysis revealed that bacterial pathogens like *P. aeruginosa* and *S. aureus* are majorly responsible for otitis media in sub-Saharan Africa. The isolates have a high level of resistance to commonly used drugs for the management of otitis media.

## Background

Otitis media is an inflammation of the tympanic membrane and middle ear with a spectrum including acute otitis media, otitis media with effusion and chronic suppurative otitis media [1, 2]. Otitis media often occur secondary to acute upper respiratory tract infections, can also be caused by allergy and changes of the middle ear or Eustachian tube anatomically or functionally [3]. Worldwide around 1.23 billion people are affected by otitis media, thus it is ranked as the fifth global burden of disease and the second cause of hearing loss [2, 4]. Children are the most affected groups and it is one of the commonest disease responsible for receiving antibiotics among children [4–6]. Otitis media is a major chronic disease in low and middle-income countries than in high-income countries. The highest incidence rate reported from Sub-Saharan Africa and South Asia [4–8].

Otitis media, especially with a chronic and recurrent form, is associated with complications such as hearing loss, decreased learning capability, and low educational achievement [9]. Around 20,000 people, majorly children under 5 years of age, die every year with the associated complications [7].

Majorly pathogenic bacteria such as non-typable *Haemophilus influenzae, Streptococcus pneumoniae, Staphylococcus aureus, Streptococcus pyogenes, Pseudomonas aeruginosa, Proteus mirabilis, Escherichia coli,* and *Moraxella catarrhalis* are the etiological agents of otitis media [10–15], although viruses and fungi also associated [14–16]. The occurrence of otitis media is directly related to the colonization rate of the nasopharynx by the bacteria [12]. Viral upper respiratory tract infections (URTI), disrupts the mucociliary system, impairs the host’s primary mechanical defense for bacterial invasion and predispose children to acute otitis media (AOM) [12–15]. This study is aimed to quantitatively estimate the isolation rate of bacteria from patients with otitis media (OM), major isolates and resistance of isolates to commonly used antibacterial agents.

## Methods

### Study protocol

Preferred Reporting Items for Systematic Review and Meta-analysis (PRISMA) flow diagram [17] was followed for identification of records, screening of titles and abstracts, and evaluation of full texts for inclusion. We have followed the PRISMA checklist [18] throughout the review and the published methodology can be accessed from http://www.crd.york.ac.uk/PROSPERO/display_record.php? ID = CRD42018102485. The study protocol is also registered on PROSPERO with reference number ID: CRD42018102485.

### Identification of records and search strategy

Legitimate databases and indexing services like PubMed/MEDLINE, EMBASE (Ovid interface), ScienceDirect and other supplementary sources, including Google Scholar, World Cat catalog, WHO African Index-Medicus and Cochrane library were visited to carry out the literature search. Relevant findings closely related to bacterial otitis media and isolation of agents were retrieved through advanced search strategies in major databases. HINARI: WHO for developing countries was used to access articles published in subscription-based journals under Science-Direct and Wiley online library. Indexing terms and carefully selected key-words were used to facilitate the search within the specified time (online records from 2009 to 2018). However, there is a deviation from the protocol published on PROSPERO, which stated from 2013 to 2018. The search was expanded back to 2009 to include more studies for subgroup analysis and to have a good pooled estimate. The time frame is set because antimicrobial resistance is a time-sensitive issue and is changing through time.

For systematic identification of records, keywords like Otitis Media [MeSH], Bacteria [MeSH], Africa South of the Sahara [MeSH], Otitis Media with Effusion [MeSH], Otitis Media, Suppurative [MeSH], “middle ear inflammation”, “middle ear effusion”, “secretory otitis media”, “purulent otitis media” and each sub-Saharan country entry term with Boolean operators (AND, OR), and truncation were used. All articles available online until February 25 – March 5, 2019, were considered. Google Scholar and World Cat were used to access gray literature from organizations and online university repositories. There was no language preference set in search strategy for all databases except for Google Scholar search, where the language preference was set to English and French. This deviation from the protocol (articles only English language) was made after observing some studies in the region that were published in the French language.

### Screening and eligibility of studies

ENDNOTE version 8.0.1 (Thomson Reuters, Stamford, CT, USA) was used to manage the search results and to identify duplicate records. Records were also checked manually to avoid duplicates due to variation in reference styles across sources followed by a screening of titles and abstracts with predefined inclusion criteria. Two authors (TT and HM) independently evaluated the full texts of selected articles for eligibility and the discrepancies between the two authors’ evaluations were solved by the other authors to come up with a consensus.

### Inclusion and exclusion criteria

Patients with clinically confirmed otitis media were the study participants. Otitis media was defined as middle ear inflammation with discharge and without a clear indication of the tympanic membrane, and participants with middle ear inflammation with discharge for more than 3 months with a perforated tympanic membrane were considered as chronic suppurative otitis media. Records, published from 2009 to 2018, on the isolation of bacterial pathogens from ear swab or discharge, and susceptibility testing were included regardless of the patient’s clinical characteristics.

Review and original articles conducted outside of sub-Saharan Africa were excluded during initial screening. Articles with data collection period before 2009, having unrelated, missing or insufficient outcomes, and irretrievable full texts (after requesting the corresponding authors) were excluded.

### Data extraction

Two authors (TT and HM) independently extracted important data related to study characteristics (country, first author, year of publication, study design, patient characteristics, number of culture-positive results (bacterial), nature of bacterial isolates, and number of isolates) and outcome of interest (number of culture-positive results, number of isolates with prevalence data for each bacterium, and susceptibility test results) using data abstraction format prepared in Microsoft Excel (Data Extraction and Summary sheet).

### Critical appraisal of studies

Critical appraisal to assess the internal (systematic error) and external (generalizability) validity of studies and to reduce the risk of biases was conducted according to the Joanna Briggs Institute’s critical appraisal tool adapted for prevalence studies [19] and graded out of 9 points. The mean score of the two authors was taken for final decision and studies with a score greater than or equal to five were included.

### Outcome measurements

The primary outcome measure is the prevalence of bacterial otitis media, major isolates and the antimicrobial resistance in sub-Saharan Africa. The measurement regarding antimicrobial resistance was conducted for selected drugs (Ampicillin, Sulfamethoxazole-Trimethoprim (cotrimoxazole), Chloramphenicol, Amoxicillin-clavulanic acid, Gentamycin, Ceftriaxone, Cefuroxime, Erythromycin, Tetracycline, and Ciprofloxacin) obtained from patients with presumed or confirmed otitis media.

### Data processing and statistical analysis

Stata version 15 was used for analyses of outcome measures after importing data extracted in Microsoft excel format. We have applied the DerSimonian and Laird’s random-effects model for the analyses at a 95% confidence level considering the variation in true effect sizes across the population (clinical heterogeneity). The heterogeneity of studies was determined using I^2^ statistics. A univariate meta-regression model was performed, with a cutoff value *p*-value< 0.05, on study characteristics to assess the possible source of heterogeneity. Begg’s and Egger’s tests were used to evaluate the presence of publication bias, and presented with funnel plots of the standard error of proportions [20, 21].

## Results

### Search results

**Fig. 1:**
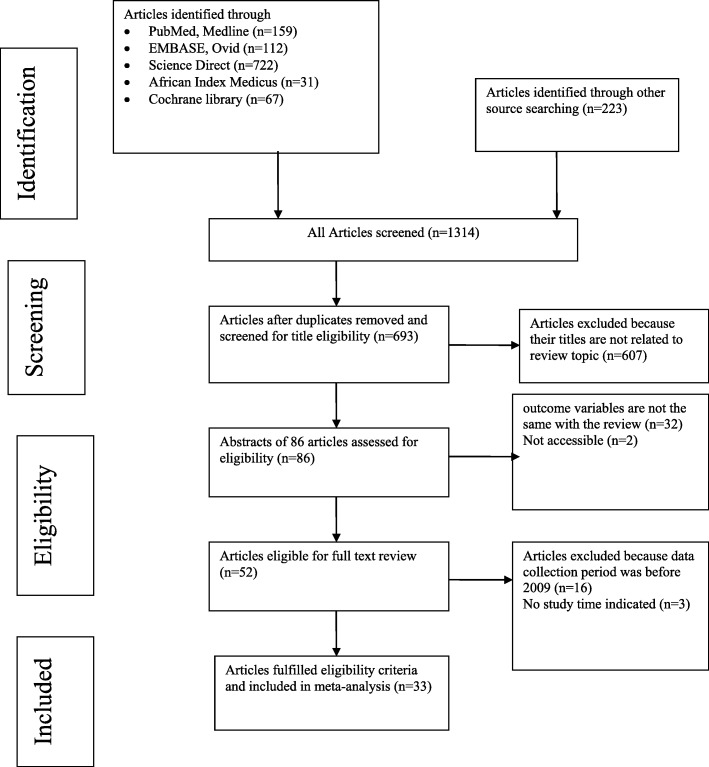
PRISMA flow diagram for Bacterial Otitis Media in sub-Saharan Africa

We conducted a systematic review and Meta-analysis per under the PRISMA statement [17]. In our literature search, a total of 1314 records were identified from several sources. From these, 621 duplicate articles were removed with the help of ENDNOTE and manual tracing. The remaining 693 records were screened using their titles and abstracts, and 607 of them were excluded. Eligibility evaluation of full texts was done for 86 records. From these, 32 articles were also excluded as the outcome of interest was missing, insufficient and/or ambiguous, and 2 articles were not accessible in full text [22, 23]. Fifty-two studies were evaluated by a full-text review and 16 studies had data collection period before 2009 and three studies [24–26] have no record of the data collection period. Finally, 33 articles passed the eligibility criteria and quality assessment and hence included in the study (Fig. 1). Thirty-two studies were utilized for the analysis of prevalence, one study has reported only the isolates without the prevalence of otitis media, and only 22 studies were utilized for the assessment of susceptibility testing since the other 11 studies have an incomplete report regarding study outcome and in some studies, the test was done with a variable number of isolates for different drugs.

### Study characteristics

As shown in Table 1, a total of 33 studies with 6034 participants were included for systematic review and meta-analysis. Seven of the included studies were retrospective analyses of secondary data (record review) from regional laboratories and hospitals [28, 29, 35, 37, 42, 43, 48]. Fourteen studies were conducted on chronic suppurative otitis media [27, 31, 33, 36, 40–42, 45, 48, 50, 53, 54, 56, 58] and the rest included studies do not indicate the type of otitis media. Seven studies [30, 32, 35, 39, 44, 52, 57] were conducted in children whereas the rest of the studies included patients of all age groups. The average quality scores of studies ranged from 5 to 8 as per the Joanna Briggs Institute scoring scale for prevalence studies (Table 1). All studies have collected ear swab/discharge samples and applied standard microbiological culturing techniques for isolation of bacterial agents and followed the international standard for performing susceptibility testing and interpretation of results (CLSI standards). Concerning the country of study, 14 studies were from Nigeria, 8 studies from Ethiopia, 2 studies from Zambia, and a single study from Angola, South Africa, Sudan, Burkina Faso, Tanzania, Uganda, Ghana, and Malawi.

**Table 1:** Characteristics of studies describing the prevalence of bacterial otitis media

Sr.No.	Author	Positive	Sample size	Country	Study design	Population characteristics	Year	Quality score	Number of isolates	Type of sample	Age of participants
1	Afolabi OA et al. (2012) [27]	134.5	135	Nigeria	CS	patients with CSOM	2012	6	138	Ear discharge	5-10 yrs. = 46; 11-20 yrs. = 26; 21-30 yrs. = 18; 31-40 yrs. = 20; 41-50 yrs. = 6; 51-60 yrs. = 10; 61-70 yrs. = 14
2	Appiah-Korang L., et al. (2014) [28]	277	315	Ghana	CS®	patients with OM	2013	5	322	Ear swab	<=1 yrs. = 64; 2-4 yrs. = 63; 5-13 yrs. = 55; 14-19 yrs. = 11; 20-44 yrs. = 53; 45-64 yrs. = 19; > = 65 yrs. = 7
3	Argaw-Denboba A et.al. (2014) [29]	1024	1225	Ethiopia	CS®	patients with OM	2014	8	1124	Ear discharge	< 5 yrs. = 153; 5-15 yrs. = 253; 16-35 yrs. = 434; 36-50 yrs. = 105; > = 51 = 47
4	Chidozie AH et al. (2015) [30]	137	156	Nigeria	CS	pediatric patients with OM	2014	7	152	Ear discharge	<=5 yrs. = 137
5	Chirwa M., et al. (2015) [31]	116	118	Malawi	CS	patients with CSOM	2013	8	214	Ear discharge	< 18 yrs. = 64; > = 18 yrs. = 40
6	Ejiofor S. O., et al. (2016) [32]	30	40	Nigeria	CS	pediatric patients with OM	2015	7	43	Ear swab	<=1 yrs. = 22; 2-4 yrs. = 8; 5-7 yrs. = 5; 8-9 yrs. = 5
7	Elmustafa M., et al. (2016) [33]	204	217	Sudan	CS	patients with CSOM	2013	5	305	Ear discharge	< 16 yrs. = 90; 16-30 yrs. = 50; 31-45 yrs. = 37; 46-60 yrs. = 31; > 60 yrs. = 8
8	Fayemiwo SA et.al. (2017) [34]	91	98	Nigeria	CS	patients with OM	2017	6	115	Ear discharge	< 10 yrs. = 54; 11-20 yrs. = 8; 21-30 yrs. = 20; 31-40 yrs. = 4; 41-50 yrs. = 3; > 50 yrs. = 9
9	Garba B. I., et al. (2017) [35]	43	53	Nigeria	CS®	pediatric patients with OM	2017	5	45	Ear swab	0–5 yrs. = 38; 5–10 yrs. = 10; 10-15 yrs. = 5
10	Habibu A. (2015) [36]	68.5	69	Nigeria	CS	patients with CSOM	2013	5	68	Ear discharge	0 – 5 yrs. = 14; 6-11 yrs. = 12; 12-17 yrs. = 8; 18-23 yrs. = 1; 24-29 yrs. = 3; 30-35 yrs. = 0; 36-41 yrs. = 0; > 41 yrs. = 1
11	Hailu D., et al. (2016) [37]	296	368	Ethiopia	CS®	patients with OM	2015	5	296	Ear discharge	0-10 yrs. = 91; 11-20 yrs. = 102; 21-30 yrs. = 51; 31-40 yrs. = 31; 41-50 yrs. = 17; 51-60 yrs. = 3; > 61 yrs. = 1
12	Jido BA et al. (2014) [38]	95	110	Nigeria	CS	Patients with OM	2011	6	95	Ear swab	0-5 yrs. = 61; 6-10 yrs. = 14; 11-15 yrs. = 5;> = 16 yrs. = 6
13	Jik A et al. (2015) [39]	154	182	Nigeria	CS	pediatric patients with OM	2015	7	154	Ear swab	1-3 yrs. = 59; 4-6 yrs. = 47; 7-9 yrs. = 30; 10-12 yrs. = 18
14	Justin R et al. (2018) [40]	89.5	90	Uganda	CS	patients with CSOM	2016	5	127	Ear discharge	< 18 yrs. = 47; > 18 yrs. = 42
15	Kazeem M. J. and R. Aiyeleso (2016) [41]	360	380	Nigeria	CS	patients with CSOM	2015	5	371	Ear discharge	< 10 yrs. = 256; 11-20 yrs. = 72; 21-30 yrs. = 26; 31-40 yrs. = 15; 41-50 yrs. = 3; 51-60 yrs. = 7; > 60 yrs. = 1
16	Matundwelo N. and C. Mwansasu (2016) [42]	60.5	61	Zambia	CS®	patients with CSOM	2016	6	154	Ear discharge	0-7 yrs. = 34; 8-15 yrs. = 26
17	Muluye D et al. (2013) [43]	204	228	Ethiopia	CS®	patients with OM	2013	7	204	Ear discharge	0-5 yrs. = 51; 6-10 yrs. = 31; 11-15 yrs. = 17; 16-20 yrs. = 21; 21-30 yrs. = 45; 31-40 yrs. = 21; > = 41 yrs. = 18
18	Nwogwugwu, NU et al. (2014) [44]	128	152	Nigeria	CS	pediatric patients with OM	2014	6	128	Ear discharge	no age specific data
19	Ogah S. and J. Ogah (2016) [45]	88	96	Nigeria	CS	patients with CSOM	2013	5	149	Ear discharge	0-10 yrs. = 34; 11-20 yrs. = 28; 21 = 30 yrs. = 16; 31-40 yrs. = 12; 41-50 yrs. = 8; 51-60 yrs. = 6; 61-70 yrs. = 2; 71-80 yrs. = 4
20	Ohieku, J. and F. Fakuade (2013) [46]	107	125	Nigeria	CS	Patients with OM	2010	5	117	Ear swab	0-2 yrs. = 49; 2-5 yrs. = 20; 6-12 yrs. = 9; 13-18 yrs. = 4; 19-64 yrs. = 24; > = 65 yrs. = 1
21	Onifade AK et al. (2018) [47]			Nigeria	CS	Patients with OM	2015	5	537	Ear discharge	
22	Orji F. T. and B. O. Dike (2015) [48]	202	206	Nigeria	CS®	patients with CSOM	2013	5	250	Ear discharge	< 1 yrs. = 12; 1-5 yrs. = 37; 5-15 yrs. = 58; > 15 yrs. = 99
23	Ouedraogo RW et al. (2012) [49]	34	41	Burkina Faso	CS	patients with OM	2012	5	36	Ear swab	no age specific data
24	Phiri H., et al. (2016) [50]	98	100	Zambia	CS	patients with CSOM	2016	5	169	Ear discharge	<=5 yrs. = 19; 6-10 yrs. = 7; 11-15 yrs. = 7; 16-20 yrs. = 15; 21-25 yrs. = 7; 26-30 yrs. = 13; 31-35 yrs. = 6; 36-40 yrs. = 8; 41-45 yrs. = 5; 46-50 yrs. = 5; 51-55 yrs. = 2; 61-65 yrs. = 1; 66-70 yrs. = 5
25	Seid A et al. (2014) [51]	171	191	Ethiopia	CS	patients with OM	2010	7	207	Ear discharge	< 5ys = 33; 5-9 yrs. = 18; 10-14 yrs. = 35; 15-19 yrs. = 32; 20-24 yrs. = 20; 25-29 yrs. = 17; 30-34 yrs. = 7; 35-39 yrs. = 10; 40-44 yrs. = 6; 45-49 yrs. = 4; > = 50 yrs. = 9
26	Tiedt NJ et al. (2013) [52]	80	86	South Africa	CS	pediatric patients with OM	2013	6	153	Ear discharge	< 13 yrs. = 86
27	Udden F., et al. (2018) [53]	150	152	Angola	CS	patients with CSOM	2016	5	443	Ear discharge	< 5 yrs. = 34; 5-9 yrs. = 23; 10-14 yrs. = 24; > = 15 yrs. = 55; no age data = 16
28	Wasihun A. G. and Y. Zemene (2015) [54]	157	162	Ethiopia	CS	patients with CSOM	2015	8	216	Ear discharge	0-5 yrs. = 30; 6-10 yrs. = 41; 11-15 yrs. = 22; 16-20 yrs. = 11; 21-25 yrs. = 23; 26-30 yrs. = 9; > 30 yrs. = 26
29	Worku S., et al. (2017) [55]	154	167	Ethiopia	CS	patients with OM	2014	7	171	Ear discharge	< 15 yrs. = 60; 15-40 yrs. = 81; > 40 yrs. = 26
30	Adoga AA et al. (2011) [56]^a^	75	97	Nigeria	CS	patients with CSOM	2009	5	75	Ear discharge	1-10 yrs. = 40; 11-20 yrs. = 23; 21-30 yrs. = 16; 31-50 yrs. = 11; > 50 yrs. = 7
31	Hailegiyorgis TT et al. (2018) [57]^a^	95	196	Ethiopia	CS	pediatric patients with OM	2014	7	95	Ear swab	< 2 yrs. = 41; 2-4 yrs. = 36; > 4-5 yrs. = 18
32	Mushi M. F., et al. (2016) [58]^a^	126	301	Tanzania	CS	patients with CSOM	2014	6	60	Ear discharge	1-10 yrs. = 31; 11-20 yrs. = 52; 21-30 yrs. = 51; 31-40 yrs. = 59; 41-50 yrs. = 55; > 50 yrs. = 33
33	Worku M. and M. Bekele (2014) [59]^a^	61	117	Ethiopia	CS	patients with OM	2013	6	69	Ear swab	Children = 53; Adult = 64

*CS* cross-sectional, *CS®* Retrospective cross-sectional, *OM* Otitis Media, *CSOM* Chronic Suppurative Otitis Media

^a^Studies removed from analysis of pooled prevalence by sensitivity analysis

### Study outcome measures

#### Prevalence of bacterial otitis media

**Fig. 2:**
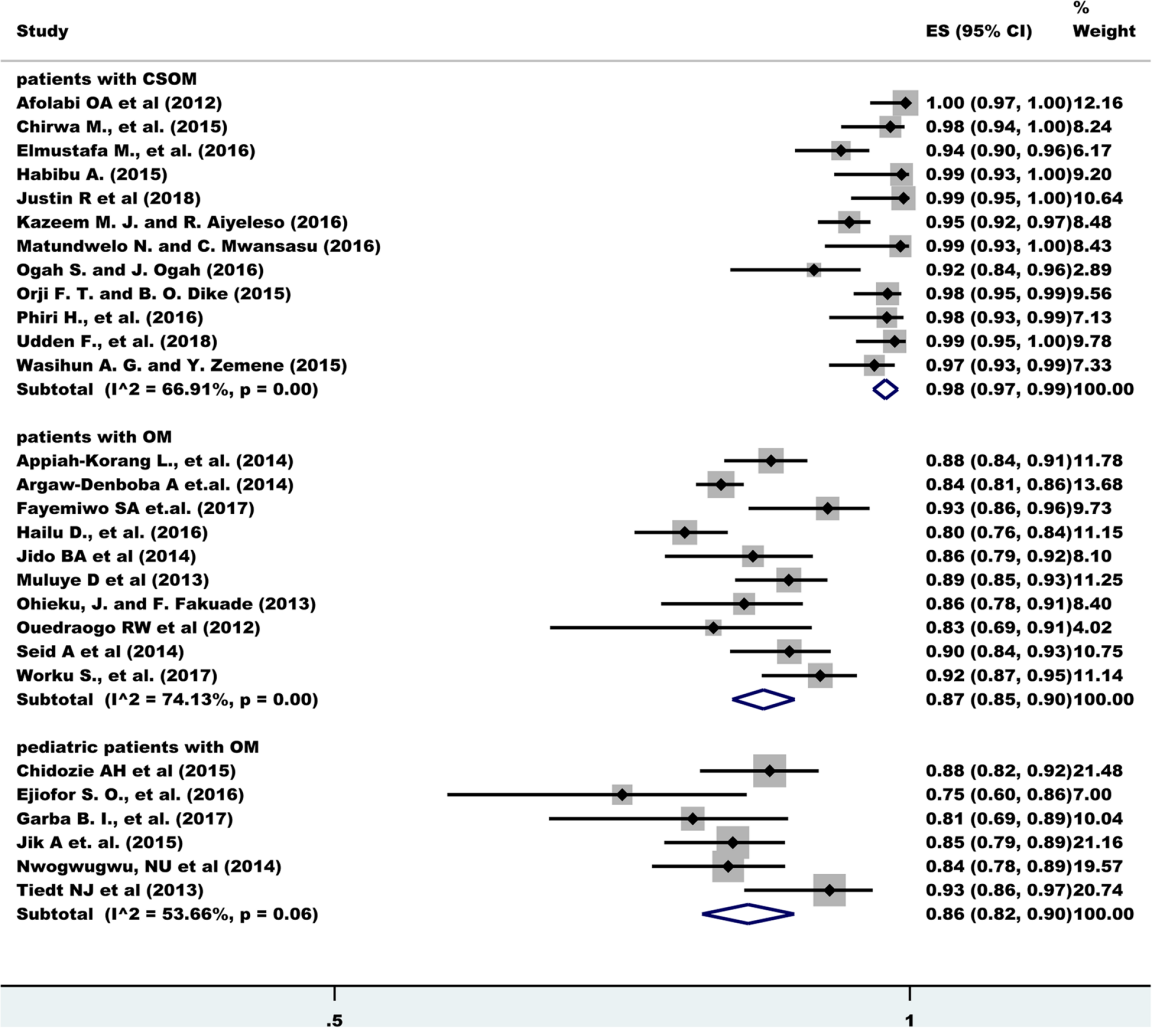
Frost plot depicting bacterial Otitis Media in sub-Saharan Africa

The overall estimate of bacterial isolation was 92% (95% CI: 90.0, 94.0) with a degree of heterogeneity (I^2^), 93.77%. A change was observed on the degree heterogeneity after excluding the known outliers and, performing subgroup analysis and sensitivity testing based on the type of otitis media and age group. The highest bacterial isolation rate was obtained among patients with chronic suppurative otitis media (CSOM) (98%) with a 66.91% degree of heterogeneity. Among patients with otitis media with inclusion of all age groups the prevalence was 87% (I^2^ = 74.13%) and among pediatric patients with otitis media 86% (I^2^ = 53.66%) (Fig. 2).

After a univariate meta-regression analysis, the year of study, country of study, study design, and sample size were not found to be statistically significant, however, the type of otitis media was found to be significant (*p*-value = 0.001).

### Bacterial isolates

From all bacterial isolates, the commonest isolate was *Pseudomonas* species (majorly *P. aeruginosa*) (ranging from 23 to 25% across subgroups) followed by *S. aureus* (18–27%), *Proteus* species (mainly *P. mirabilis*), *Klebsiella* species, Coagulase Negative *Staphylococci* (CoNS) and *Streptococcus* species like *S. pyogenes*, and *Enterococcus* species. Other gram-negative rods under the *Enterobacteriaceae* (*Citrobacter* species, *Providencia* species, *Serratia* species, *Enterobacter* species, and *Morganella* species) account for 7–11%, but separately they account for less than 5% (Table 2, and Data Extraction and Summary sheet: *isolates*). On the subgroup analysis, *S. aureus* was found to be the major isolate except for chronic suppurative otitis media and *Streptococcus* species were observed to have a relatively higher prevalence in pediatric patients with otitis media (Table 2).

**Table 2:** Subgroup analysis of bacterial isolates by type of otitis media

Bacterial isolate	Type of otitis media, prevalence % (95% CI)								
	Patients with CSOM			Patients with OM			Pediatric patients OM		
	Pooled prevalence (%)	No of study	I^**2**^ (%)	Pooled prevalence (%)	No of study	I^**2**^ (%)	Pooled prevalence (%)	No of study	I^**2**^ (%)
*S. aureus*	18(11–25)	14	96.2	23(19–27)	12	88.29	27(15–39)	7	94.9
CoNS	7(2–12)	4	82.5	9(4–14)	6	96.98	1(0–5)	1	
*Streptococcus* spp	5(3–7)	10	79.18	2(1–4)	8	70.79	10(4–15)	5	82.7
*Pseudomonas* spp	23(18–29)	14	92.47	24(18–29)	12	93.95	25(16–33)	7	88.33
*Proteus* spp	19(15–22)	12	81.00	19(14–23)	12	89.68	11(5–18)	6	90.29
*E. coli*	9(6–11)	13	88.04	7(3–10)	11	94.06	8(5–11)	7	57.47
*Klebsiella* spp	12(9–16)	12	91.15	7(5–10)	11	91.01	5(1–9)	4	82.95
Other Enterobacteriaceae	10(2–17)	5	95.73	11(6–17)	6	92.31	7(1–14)	3	
*S. pneumoniae*	3 (1–6)	4	88.29	2 (1–4)	5	92.87	6 (3–9)	4	39.46
*H. influenzae*	2 (0–3)	2		1 (0–3)	1		8(5–11)	2	

Four studies have demonstrated the isolation of *Acinetobacter* species, and *Corynebacterium* species were isolated by two studies. Anaerobic bacteria were not commonly isolated from the studies, but a study by Chirwa M et al [31] has reported the isolation of *Bacteroides* species and *Peptostreptococcus* species.

Though the main objective of this review is bacterial etiologies we found out that fungal pathogens like *Candida* species and *Aspergillus* species account for 6% (3–8%) of the etiology for otitis media; only one study has indicated *Aspergillus* species.

### The resistance of isolates to antibacterial agents

Studies have included various drugs for susceptibility testing. We have selected drugs that have been tested in more than 5 studies and accordingly, the analysis was done for Ampicillin, Cotrimoxazole, Cefuroxime, Amoxicillin*-*clavulanate, Ciprofloxacin, Gentamycin, Chloramphenicol, Amoxicillin, Ceftriaxone, Tetracycline, and Erythromycin.

A higher degree of pooled resistance was observed in Ampicillin (72%, ranging from 69 to 94% across subgroups), Amoxicillin (68, 62–85%), Cotrimoxazole (60, 41–90%), Amoxicillin*-*clavulanic acid (54, 47–61%), and Cefuroxime (51, 17–64%) and. The lowest degree of resistance was found among Ciprofloxacin, Gentamycin, and Chloramphenicol (Table 3, and Data Extraction and Summary sheet: *susceptibility*). Generally, a higher degree of resistance was observed in pediatric patients with otitis media than the other subgroups.

**Table 3:** Subgroup analysis of drug resistance by type of otitis media

Bacterial isolate	Type of otitis media, prevalence % (95% CI)								
	Patients with CSOM			Patients with OM			Pediatric patients OM		
	Pooled prevalence (%)	No of study	I^**2**^ (%)	Pooled prevalence (%)	No of study	I^**2**^ (%)	Pooled prevalence (%)	No of study	I^**2**^ (%)
Ampicillin	70(54–86)	8	98.56	69(48–90)	7	99.09	94(91–98)	2	
Cotrimoxazole	63(47–80)	7	98.03	41(11–72)	6	99.58	90(78–100)	3	
Amoxicillin	–	–	–	62(37–87)	5	98.78	85(81–89)	2	
Ceftazidime	52(28–76)	4	97.09	7(1–16)	3		57(47–66)	1	
Chloramphenicol	32(21–48)	8	95.98	25(13–38)	6	97.87	55(45–64)	1	
Ciprofloxacin	19(12–25)	10	94.36	16(9–24)	6	94.23	34(12–56)	3	
Gentamycin	23(15–31)	11	96.54	26(13–40)	6	98.25	55(34–75)	4	92.92
Amoxicillin- Clavulanic acid	53(32–74)	7	98.76	47(8–86)	4	99.48	61(13–100)	4	99.59
Erythromycin	41(16–66)	6	98.91	38(6–71)	5	99.43	81(68–93)	3	
Cefuroxime	64(60–69)	2		17(14–20)	2		60(27–93)	4	98.08
Ceftriaxone	32(18–46)	8	97.73	18(10–26)	5	91.71	75(58–92)	4	92.19
Tetracycline	37(10–63)	4	98.85	20(6–35)	3		98(88–100)	1	

### Publication bias

**Fig. 3:**
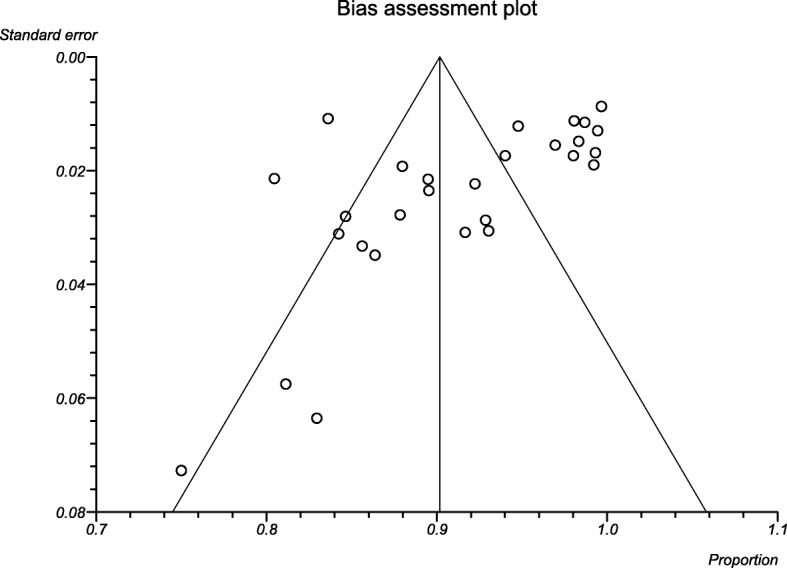
Funnel plot depicting publication bias for Bacterial Otitis Media in sub-Saharan Africa

There is some evidence of publication bias in studies reporting the prevalence of bacterial isolates as confirmed by funnel plots of the standard error with proportion supplemented by statistical tests (Begg’s test, *p* = 0.0016; Egger’s test, *p* = 0.0211) (Fig. 3). However, there was no significant publication bias in the subgroup analysis.

## Discussion

This analysis included 32 original studies addressing bacterial isolation rate from otitis media within the specified time-frame. Since all the included studies have a cross-sectional study design the pooled analysis could be used to assess the relative importance of various pathogens in Africa as well as worldwide. All the included studies in this review have collected an ear swab/discharge sample for microbiological analysis. The bacterial isolation rate using culture-based techniques ranged from 86% (pediatric patients) to 98% (patients with CSOM). Data coverage was featuring predominantly within some countries like Nigeria while most other countries don’t have any study to represent the population.

Bacteria can reach the middle ear in otitis media from nasopharynx through the Eustachian tube or from the external ear canal through the non-intact eardrum. Aerobic bacteria like *S. aureus*, *P. aeruginosa* and *Proteus* species (majorly *P. mirabilis*) were the main bacterial pathogens for otitis media in this analysis. The finding of those pathogens as a major isolate provides a more definitive confirmation of what has been reported by other published single-country studies as the result consistent [37, 41, 45]. Other bacteria are also indicated from studies like *Klebsiella* species (6–11%), CoNS (6–12%), and *E. coli* (5–9%).

The prevalence of other pathogens like *H. influenzae*, *S. pneumoniae* and, *M. catarrhalis* is very minimal, even though those isolates were indicated from another review [60, 61], and commonly reported from AOM and OME episodes, especially during childhood [4, 12, 14]. Since most of the studies conducted in sub-Saharan Africa are on CSOM and the collected samples are ear swab/discharge these pathogens are less likely to be reported from the studies. This minimal finding could also be due to regional differences or the absence of studies conducted using molecular techniques that had higher sensitivity compared to culture techniques.

On a recent international consensus on the management of otitis media with effusion panelists generally agreed not to consider antibiotics as a non-surgical management option for OME [62]. Several other reviews have indicated that the management of OM is far better with antibiotics and oto-topical agents than without any treatment, especially if it is CSOM [63, 64]. Considering this review setting and population, treatment with antibiotics is a primary option as most of the studies are conducted on patients with CSOM. On the contrary, isolates from otitis media were having a high level of resistance to commonly used antibacterial agents in this analysis. The highest resistance is against ampicillin (ranging from 69 to 94%) followed by Amoxicillin (62–85%), cotrimoxazole (41–90%), Augmentin (47–61%) and Cefuroxime (17–64%). Generally, the resistance to erythromycin, ceftriaxone, Tetracycline, and ceftazidime was less than 50% for most of the studies. Those drugs are commonly used drugs for the management of otitis media, especially in low-income country settings without appropriate microbiological diagnostic service. This review also found that relatively higher susceptibility of isolates to ciprofloxacin, gentamycin, and chloramphenicol, less than 35% resistance. The studies included in this review revealed that non-susceptibility to fluoroquinolones ranged between 16 and 34%.

All studies in this review span on the analysis of ear swab/ discharge, which is a late-onset scenario. Early diagnosis and management of otitis media have a paramount effect on the health of the patients, especially children, as it has complications ranging from tympanic membrane perforation to neurological impairments. Early-onset infections will require the collection of middle ear fluid by tympanocentesis, which will be hard to practice in sub-Saharan Africa, without advanced medical facilities. A clear contribution of pathogens, like fastidious and anaerobic bacteria, can be understood by using highly sensitive laboratory techniques.

It is unlikely that this review has missed a relevant study as we have conducted a highly sensitive systematic search, including grey literature. The dearth of appropriate population-based elegant studies, risk of bias in prevalence studies, mainly due to under-reporting; and heterogeneity of data were the main limitations of this review. Etiological data were more consistent with some discrepancies in isolation rates, which can be explained by the quality of the microbiological procedures used, such as sample collection and transportation and bacterial isolation in some studies. One of the recognized limitations in such pooled studies [65] is differences in middle ear fluid collection guidelines in different localities even with efforts to standardize the design across studies. There are likely differences in care-seeking practices for OM among countries, affecting the severity of cases, and one country may collect middle ear fluid only in cases considered to be recurrent OM. The true contribution of these bacteria to OM is likely higher than the reported as bacterial etiology was not determined by molecular techniques in the included studies [66]. Different clinical severity or age tropisms may have been observed from culture-negative samples with molecular testing.

Following accepted methods, all-inclusive searching with date range, put together with a quality appraisal of the included studies are the main strength of this review. Stakeholders who focus on such matters will get a great benefit from compiling all available evidence to make informed decisions on prevention measures.

## Conclusions

The analysis revealed that *P. aeruginosa, S. aureus* and *P. mirabilis* are the commonest bacterial pathogens responsible for otitis media in sub-Saharan Africa. A high level of resistance was observed to commonly used antibacterial agents such as Ampicillin, cotrimoxazole, Amoxicillin, and Amoxicillin-clavulanate however, isolates were less resistant to Ciprofloxacin, Gentamycin, and Chloramphenicol.

Thus, drugs like Ampicillin, amoxicillin, Amoxicillin-clavulanate, and cotrimoxazole should not be used as a first-line treatment in sub-Saharan countries since countries in this geographical location are commonly dependent on clinical data for treatment with the absence of microbiology laboratory. Without proper treatment, otitis media could lead to intracranial and intratemporal complications with higher and complex management.

## Supplementary information

**Additional file 1:**Data extraction and summary sheet. **Sheet 1 (character):** participant information and culture positive rate of Otitis media. **Sheet 2 (isolates):** Bacterial isolates from Otitis media. **Sheet 3 (susceptibility):** Drug susceptibility test of isolates.

## Data Availability

All data generated or analyzed during this study are included in this published article as supplementary information files.

## Abbreviations

AOM: Acute Otitis Media
CoNS: Coagulase Negative Staphylococci
CSOM: Chronic Suppurative Otitis Media
OM: Otitis Media
OME: Otitis Media with Effusion
URTI: Upper Respiratory Tract Infections
WHO: World Health Organization
